# From drugs to biomaterials: a review of emerging therapeutic strategies for intervertebral disc inflammation

**DOI:** 10.3389/fcimb.2024.1303645

**Published:** 2024-01-30

**Authors:** Shuhan Yang, Shaoze Jing, Shanxi Wang, Fajing Jia

**Affiliations:** ^1^Department of Orthopedics, Third Hospital of Shanxi Medical University, Shanxi Bethune Hospital, Shanxi Academy of Medical Sciences, Tongji Shanxi Hospital, Taiyuan, China; ^2^Department of Spine Surgery, Honghui Hospital, Xi’an Jiaotong University, Xi’an, China; ^3^Department of General Practice, Third Hospital of Shanxi Medical University, Shanxi Bethune Hospital, Shanxi Academy of Medical Sciences, Tongji Shanxi Hospital, Taiyuan, China

**Keywords:** intervertebral disc, infection, inflammation, low back pain, treatment

## Abstract

Chronic low back pain (LBP) is an increasingly prevalent issue, especially among aging populations. A major underlying cause of LBP is intervertebral disc degeneration (IDD), often triggered by intervertebral disc (IVD) inflammation. Inflammation of the IVD is divided into Septic and Aseptic inflammation. Conservative therapy and surgical treatment often fail to address the root cause of IDD. Recent advances in the treatment of IVD infection and inflammation range from antibiotics and small-molecule drugs to cellular therapies, biological agents, and innovative biomaterials. This review sheds light on the complex mechanisms of IVD inflammation and physiological and biochemical processes of IDD. Furthermore, it provides an overview of recent research developments in this area, intending to identify novel therapeutic targets and guide future clinical strategies for effectively treating IVD-related conditions.

## 1 Introduction

### 1.1 IVD, IDD, and LBP

Low back pain (LBP) is a common clinical condition that has become increasingly prevalent, with an increasing incidence rate in older age groups. According to statistical reports, the global incidence rate of LBP ranges between 13.1% and 28.5% (Maher et al., 2017). Individuals from lower- and middle-income groups experience LBP less frequently than those in higher-income groups (Hartvigsen et al., 2018; Knezevic et al., 2021). LBP is a leading cause of productivity loss and a major contributor to disability worldwide, placing a significant strain on healthcare systems and global economies worldwide (Luo et al., 2004; Katz, 2006; Freburger et al., 2009).

**Figure 1:**
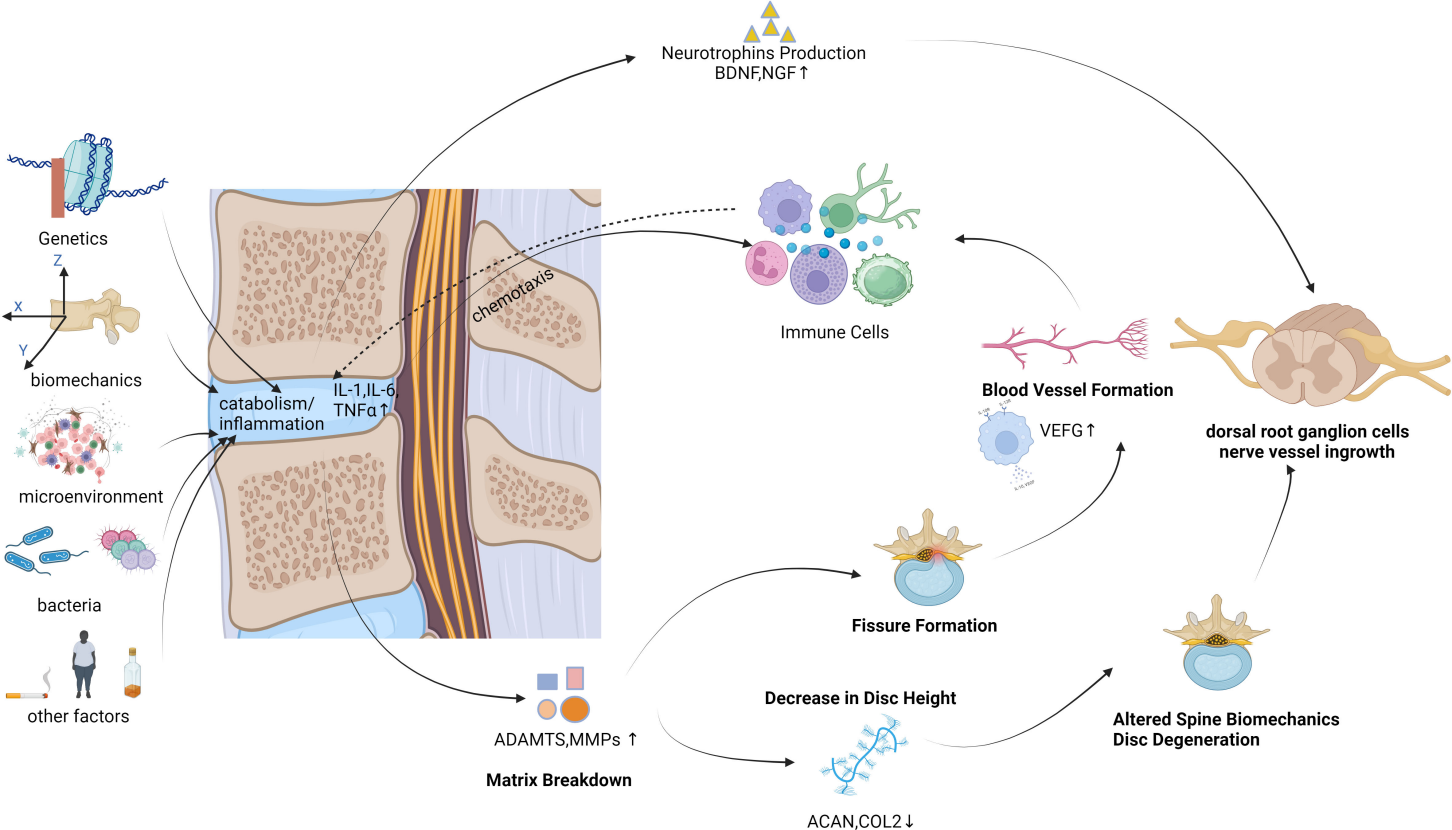
Various factors contribute to IDD, which in turn leads to LBP.

The intervertebral disc (IVD) is a sealed structure situated between the vertebral bodies of the human spine. It consists of cartilage plates, fibrous rings, and a nucleus pulposus (NP) (Gantenbein et al., 2023). The IVD serves to connect adjacent vertebral bodies and facilitate spinal movement (Nixon, 1986; Stokes and Iatridis, 2004). More than 80% of people aged above 50years experience intervertebral disc degeneration (IDD), which is commonly linked to LBP (Antoniou et al., 1996). Several pathological changes are associated with IDD, such as extracellular matrix (ECM) degradation, inflammation, and cell loss through apoptosis (Sakai and Grad, 2015). Secretion of cytokines (tumor necrosis factor-alpha [TNF-α], interleukin [IL]-1β, and IL-6 being the most prominent cytokines) leads to recruitment of host immune cells (macrophages, neutrophils, and T cells) if IVD structural defects are present. As the inflammatory response progresses, immune cells and nociceptive nerve fibers from the dorsal root ganglia begin to infiltrate the damaged IVD tissue. Upon the release of neurotrophic factors by both NP cells and immune cells, the activation of nociceptive nerve fibers occurs, initiating the process of pain transduction. Moreover, the presence of inflammatory cytokines has been found to augment degenerative mechanisms through the activation of ECM breakdown proteins and the inhibition of ECM structural molecule expression (Sun et al., 2022). The causes of IDD are diverse, encompassing genetics; biomechanical changes; alterations in the cellular microenvironment; bacterial or microbial infections; and lifestyle factors such as smoking, alcohol consumption, and obesity. Throughout the IDD process, an increase in pro-inflammatory cytokines within IVD cells is observed, culminating in cell degeneration and necrosis, ultimately leading to ECM degradation (Roughley, 2004; Adams et al., 2009; Kepler et al., 2013; Lin et al., 2023). This, in turn, brings about alterations in the structure and biomechanical properties of the spine. The increased presence of pro-inflammatory cytokines exacerbates the inflammatory response, triggers angiogenesis and neural ingrowth, and prompts the release of pain mediators within the IVD (**Figure 1**). IDD constitutes a complex and multifactorial process. Inflammation, with or without microbial infection, plays an important role in IDD (Cazzanelli and Wuertz-Kozak, 2020). This inflammation is one of the differentiating factors between symptomatic and asymptomatic IDD, indicating a connection between inflammation and LBP.

In clinical treatment (Chou et al., 2009), doctors assess patients’ symptoms and medical history based on the etiology and pathogenesis of discogenic LBP to determine the appropriate treatment approach. Common treatments include conservative treatment and surgical intervention. Conservative treatment methods are rest, physical therapy, medication, and rehabilitation exercises for pain relief, functional improvement, and promoting recovery. Surgical treatment is often recommended for patients who are nonresponsive to conservative treatment or have severe conditions that may involve procedures such as discectomy, disc replacement, or artificial disc implantation.

Research in basic medical sciences and translational medicine aims to understand the pathogenesis of discogenic LBP and to develop new treatment methods. Studies have examined the structural and histological characteristics of IVDs, along with associated molecular biology and biomechanical changes, using animal models and cell experiments. These investigations help to clarify the mechanisms of IDD and offer a theoretical foundation for new treatment strategies. Research also focuses on the development of novel diagnostic methods and therapeutic approaches. For example, researchers are exploring the use of biomarkers, imaging techniques, and genomics to diagnose discogenic LBP (Bhujel et al., 2022). Technological advancements in fields such as stem cell and gene therapy also show promise for more effective treatment options for patients (Novais et al., 2021).

This review elucidates the underlying inflammatory processes of aseptic and septic inflammation during IDD and introduces various novel drugs, materials, and interventions that can mitigate inflammation, delay the onset and progression of IDD, and alleviate patient pain. We hope that the insights provided in this review will offer new avenues for the clinical treatment of LBP related to IVD infection.

### 1.2 Physiological and biochemical changes in IDD

At present, specific blood markers are not implicated in IDD. Clinical identification of IDD relies predominantly on evaluating clinical history, imaging studies, and neurophysiological tests (Wu et al., 2020) (**Table 1**). Patients often seek medical attention when experiencing persistent lower back and leg pain, signifying the progression of IDD to the middle or late stage. Detecting early IDD poses challenges, as the loss of proteoglycans, Aggrecan, and alterations in certain proteinase levels are not readily discernible microscopically (Knezevic et al., 2021). An innovative quantitative MRI (qMRI) technique has been validated to identify specific characteristic changes associated with IDD at an earlier stage (Russo et al., 2023). In a systematic review conducted by Fabrizio Russo and colleagues (Russo et al., 2023), qMRI technology demonstrated superior efficacy compared to conventional MRI techniques in revealing subtle alterations in water content, proteoglycans, glycosaminoglycans, and select degradation markers, thereby enhancing its capability for the early detection of IDD.

**Table 1:** Clinical methods commonly used to detect intervertebral disc degeneration.

diagnostic method	advantage	disadvantage
Pain induction experiment	convenient、fast	low security、low accuracy
X-ray	economical、convenient	limited soft tissue contrast
CT	clear visualization of the intervertebral disc structure	limited soft tissue contrast
MRI	clear visualization of the intervertebral disc and its surrounding soft tissues	time-consuming, expensive
MRS	assess the content of different chemicals in the intervertebral disc	technology is not mature
qMRI	allows for early detection of changes in the substance content within the IVD	lacks uniform diagnostic criteria, technology is not mature
electrophysiological scan	testing the nerve function around the IVD	difficult to detect in the early stages
bone scan	detecting the bone structure of the IVD	radioactive, difficult to detect in the early stages

IVD is a natural aging process characterized by a recurring cycle from cell death to matrix remodeling (Kang et al., 2023). Distinguishing the pathological changes between a normally aging intervertebral disc and one affected by degeneration proves challenging (Francisco et al., 2022). Additionally, the pathophysiological alterations in IDD and the radiographic features of various clinically relevant spinal disorders are distinct (Teng et al., 2023). If radiological evidence indicates disc issues, such as disc narrowing or disc protrusion, patients often present with pain as their primary complaint. However, the presence of IDD alone does not always correlate with pain. Despite this, a strong association persists between IDD and LBP (Yang et al., 2022a).

The IVD undergoes numerous physiological and biochemical changes during normal aging or when exposed to factors such as acute trauma, bacterial infection, and gene mutations (Francisco et al., 2022). One of the most notable alterations in intervertebral disc degeneration is the reduction in Aggrecan (proteoglycan) content. Aggrecan, the most abundant proteoglycan in the IVD, experiences diminished levels, leading to the breakdown of the matrix structure (Mohd Isa et al., 2022). Consequently, this results in a reduction in disc height and static water pressure, potentially causing damage to a spinal segment if left unaddressed. Additionally, during the process of intervertebral disc degeneration, there is an increase in the content of fibrous connective proteins, further depleting the levels of Aggrecan. Simultaneously, the levels of enzymes associated with Aggrecan metabolism also undergo changes (Wang et al., 2023).

While collagen content has been observed to increase during the detection of IDD, the proportion of its subtypes undergoes alterations. Type II collagen is the predominant collagen in NP tissue; however, in IDD, the content of cross-linked and denatured type II collagen gradually decreases. Consequently, the ratio of type I collagen to type II collagen rises, leading to reduced fluidity and increased rigidity of the IVD at this stage (Trefilova et al., 2021).

Cell apoptosis is another phenomenon associated with IDD. Unfortunately, apoptosis is an irreversible event that occurs with aging, and reports indicate that, at a specific age, half of the cells in the IVD have already undergone apoptosis (Trout et al., 1982). Research has demonstrated that notochordal cells play a role in the recovery of IDD. In certain animal models of disc injury, the population of notochordal cells persists even as the animals mature (Li et al., 2023a). However, studies have shown that, even in the early stages of human development, there is a significant reduction in viable NP progenitor cells with age. Hence, many researchers believe that the loss of viable NP progenitor cells is a central factor in IDD (Harfe, 2022).

The decline in proteoglycan content constitutes another crucial aspect of IDD. The hydrated matrix tissue is predominantly composed of proteoglycans, and their reduction can impact the comprehensive physiological functions of IVD. This encompasses static water pressure, disc height, biomechanical characteristics, and more, thereby influencing the physiological activities and nutritional metabolism of the vertebral body (Silagi et al., 2018). When proteoglycans are depleted in IVD, the normally elastic fibrous tissue of the disc becomes stiffer, resulting in the generation of unnatural mechanical stress. With the loss of the hydrated matrix, the flexibility and static water pressure of the tissue gradually decrease. The sustained inward pressure on the fibrous ring leads to compression and bulging of the IVD (Peng et al., 2023). Following this, alterations in mechanical stress affect the surrounding fibrous tissue and ligaments, resulting in the thinning of IVD joints and narrowing of the spinal canal. This process persists, leading to IVD rupture and the development of advanced spinal canal stenosis (Kushchayev et al., 2018). Damage to a single spinal segment extends to involve adjacent segments, triggering substantial tissue remodeling in the spine. Additionally, the depletion of hydration in IVD matrix disrupts fluid flow and reduces the capacity to transport essential nutrients, thereby intensifying IDD and hindering regeneration (Yang et al., 2020).

### 1.3 Inflammation in IVD

**Figure 2:**
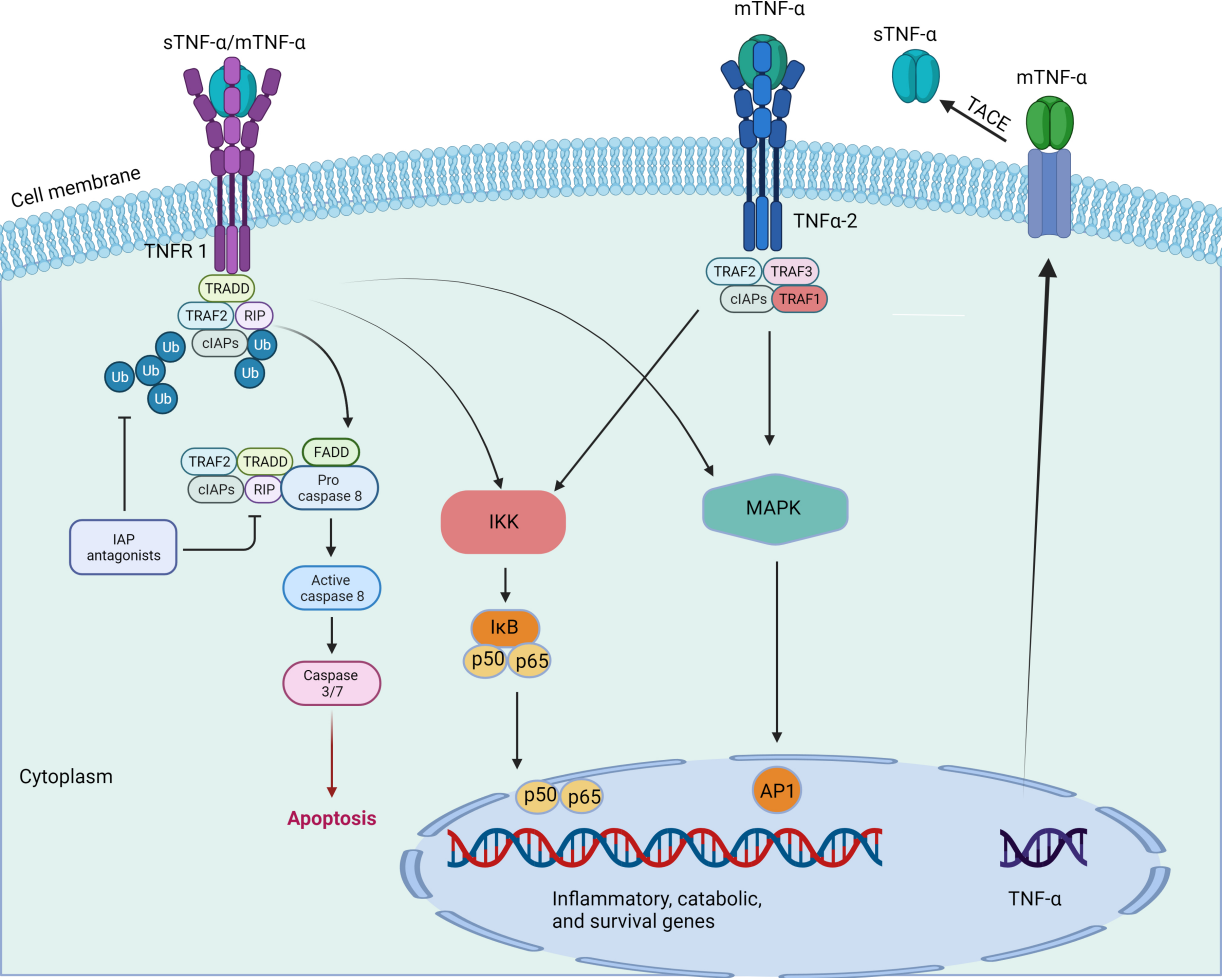
TNF-a signaling pathway.

**Figure 3:**
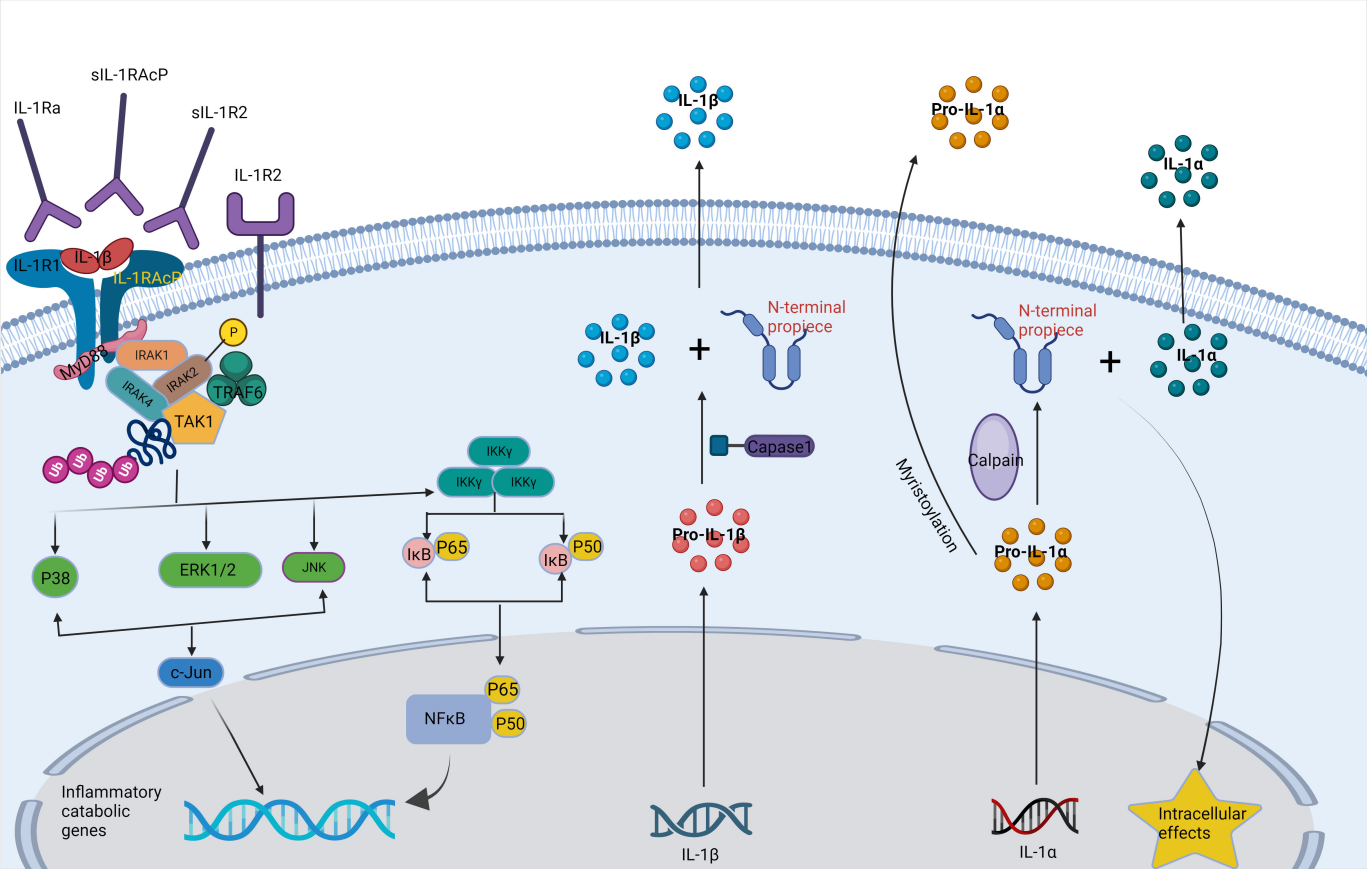
IL-la and IL-l synthesis and signal transduction pathways.

Inflammation is a defensive response of living tissues with a vascular system to damaging factors. It is also a defensive response of the body to stimuli, characterized by redness, swelling, heat, pain, and impaired function. There are many causes of inflammation in body tissues. Inflammation can be categorized into two main groups: infectious inflammation and aseptic inflammation. When the human body is infected by pathogenic microorganisms and bacteria, viruses, protozoa and other infections, and the body produces inflammatory responses such as oozing, necrosis and hyperplasia; this is collectively known as Septic inflammation. If the inflammatory reaction is caused by physical and chemical factors, they are collectively called Aseptic inflammation (Hodges et al., 2021). At the onset of inflammation, whether septic or aseptic inflammation, a large number of inflammatory factors are recruited. Many inflammatory mediators and their associated signaling pathways have critical roles in the onset and progression of IDD (Cazzanelli and Wuertz-Kozak, 2020; Zhao et al., 2021). Pro-inflammatory effects: Inflammatory factors such as TNF-α, IL-1β, and IL-1α (**Figures 2**, **3**) exert pro-inflammatory effects (Li et al., 2023b). These factors stimulate inflammatory responses and cause changes in IVD tissues (Zhang et al., 2021). Their release can lead to pain, vasodilation, and the influx of inflammatory cells (Chen et al., 2022b). Cell apoptosis: Overproduction or excessive accumulation of inflammatory factors may lead to apoptosis (cell death) (Yang et al., 2020; Chen et al., 2022b) or programmed cell death, in IVD cells. This process contributes to degenerative changes and structural damage in IVD tissues, particularly in the NP, during IVD inflammation (Zhao et al., 2021). Cell signaling of cytokines: Inflammatory factors can activate the expression of related genes through cellular signaling pathways, such as the nuclear factor-kappa B (NF-κB) pathway (Zhang et al., 2021). Elevated gene expression can worsen inflammatory reactions and accelerate disease progression (Xia et al., 2019). Induction of neuropathological changes: The release of inflammatory factors can also cause neuropathic changes, including increased pain (Wiet et al., 2017). Research indicates that inflammatory factors such as TNF-α can directly stimulate nerve endings, enhancing pain transmission and leading to neuropathological modifications. Additionally, the presence of certain inflammatory mediators promotes nerve growth into the IVD, increasing susceptibility to LBP (Mohd Isa et al., 2022).

IDD is a pathological degenerative condition affecting the IVD, a connective tissue located between the vertebrae that is essential for spinal kinematics. This degeneration occurs at the tissue, cellular, and molecular levels, resulting in significant alteration of the IVD’s structure and function. This eventually reduces its ability to tolerate compressive loads. Among the various pathological changes in IVD that are associated with IDD, the most common are degradation of the ECM degradation, inflammation, and cell loss through apoptosis (Clouet et al., 2009).

During IVD inflammation, NP cells release increasing amounts of pro-inflammatory cytokines, which can lead to progressive degeneration and the development of pain in the IVD. Among them, TNF-α, IL-1β, IL-6, and IL-17 are particularly prominent. These cytokines are known to facilitate matrix degradation, activate host immune responses, and result in the infiltration of immune cells and nerve fibers. Neural infiltration is particularly noteworthy, as it is a key source of pain in degenerative disc disease (DDD) (Risbud and Shapiro, 2014).

ECM degradation, apoptosis, and inflammation are recognized as the main characteristics of DDD. These processes are interconnected and mutually dependent (Urban and Roberts, 2003). Pro-inflammatory cytokines cause dysregulation of ECM metabolism by upregulating the expression of ECM-degrading enzymes and downregulating the expression of ECM structural components (Wuertz and Haglund, 2013; Risbud and Shapiro, 2014). This internal degradation of the ECM leads to an accumulation of ECM fragments outside the cells, further amplifying the inflammatory response of NP cells (Quero et al., 2013).

## 2 Treatment of septic inflammation

The question of whether bacterial infections contribute to IDD is still debatable. However, some patients with chronic LBP have reported relief following antibiotic treatment by alleviating pain and reducing disability (Albert et al., 2013). Stirling et al. (Stirling et al., 2001) first introduced the notion of IDD being linked to infection. They found serological evidence of Gram-positive infection in 31% of patients with radicular symptoms and “sciatica” due to herniated NP. Furthermore, they discovered that 53% of the cultured disc samples from patients who underwent microdiscectomy tested positive for bacteria, with the most commonly isolated bacteria being *Propionibacterium acnes* (*P. acnes*) *and* coagulase-negative staphylococci. He et al. reported an increase in nod-like receptor protein 3 (NLRP3) positive cells in NP tissues infected with *P. acnes*, a bacterium associated with acne. Administering the NLRP3 inhibitor MCC950 reduced the levels of inflammatory mediators IL-1β and IL-18 (He et al., 2020).

The administration of antibiotics serves multiple purposes in the context of disc surgery. Not only do they aim to prevent infections from *P. acnes* during disc surgery, but they also protect against other opportunistic pathogens that may be present on or within the host skin, in anatomical areas of interest, or within the operating room environment. Guidelines on surgical prophylaxis from the American Society of Health-System Pharmacists and the North American Spine Society recommend the use of cefazolin, a negatively charged antibiotic, as a first-line prophylactic antibiotic for patients undergoing spinal surgery in the United States. Alternative antibiotics such as clindamycin, vancomycin, and gentamicin (positively charged antibiotics) are used in specific scenarios, such as when patients have hypersensitivity to cephalosporins or β-lactams and can also be used for surgical prophylaxis (Bratzler et al., 2013; Shaffer et al., 2013). Clindamycin is usually recommended as a second-line antibiotic if the patient is allergic to penicillin or is particularly allergic to cephalosporins. Vancomycin is generally indicated for methicillin-resistant *Staphylococcus aureus* (MRSA) carriers, patients suspected of MRSA infection, or patients allergic to clindamycin. Gentamicin, which primarily targets gram-negative bacteria, is less commonly used as a prophylactic antibiotic due to its relatively high risk. Although vancomycin is an effective antibiotic, it should be used only when necessary, partly to minimize the risk of resistance development and to cope with its higher risk than that with cefazolin (Urquhart et al., 2021).

Ozone is not an antibiotic. However, it can kill a wide range of micro-organisms. Ozone therapy involves the administration of ozone directly into the affected disc location, typically performed under the administration of a local anesthetic. Ozone gas has the ability to eliminate bacteria, fungi, and viruses within contaminated discs. The process of inhibiting the growth and proliferation of microorganisms is achieved through the destruction of their cell walls or membranes. Additionally, it has the capability to impede the synthesis of inflammatory mediators and diminish the infiltration of cells involved in inflammation. Ozone has the potential to facilitate the repair and healing mechanisms of IVD. The intervention results in an augmentation of oxygen delivery to the intervertebral disc, eliciting a stimulation of cellular proliferation and production of extracellular matrix components, ultimately facilitating the process of tissue healing. Francesco Somma conducted a study wherein the Oswestry Disability Index (ODI) showed a substantial reduction among patients with disc herniation who had previously been infected with the novel coronavirus following ozone therapy (Somma et al., 2022).

The effectiveness of antibiotics in treating IVD infection or degeneration remains controversial. Lars Christian Haugli Bråten et al. conducted a double-blind, multicenter trial involving 180 patients selected from outpatient clinics in six Norwegian hospitals. Patients were randomly assigned to receive either oral amoxicillin thrice daily or a placebo for 3 months. Using the Roland-Morris Disability Questionnaire (RMDQ), patient outcomes were assessed at 1-year follow-up. The results showed that the average RMDQ score in the amoxicillin group was 1.6 points lower than that in the placebo group, but it did not reach the minimum clinically significant difference of 4 points. Therefore, their findings do not support the use of antibiotics for improving patients’ conditions in LBP treatment (Bråten et al., 2019). Further research is needed to determine the clinical efficacy and underlying mechanisms of antibiotic treatment for IVD infection.

## 3 Treatment of aseptic inflammation

### 3.1 Small molecule drug therapy

Within the realm of pharmaceuticals, small molecules are characterized as substances that possess the ability to bind to certain biological macromolecules, hence exerting influence over distinct biological processes. Small molecules exhibit a maximum molecular weight of 900 Daltons and necessitate efficient diffusion across cellular membranes and the digestive system in order to be absorbed. In general, molecules with a molecular weight exceeding 550 Daltons have greater difficulties in terms of absorption, although certain molecules with a molecular weight of up to 900 Daltons have been observed to effectively traverse the barrier (Hojjat-Farsangi, 2014; Sandborn, 2015). The utilization of small molecules as medicinal agents offers numerous advantages. Due to their diminutive dimensions, they provoke a reduced immunological response within the host and are believed to possess properties that counteract inflammation, apoptosis, and oxidative stress while also exhibiting anabolic and anti-catabolic activities (Kamali et al., 2021). Certain small compounds have demonstrated encouraging outcomes as alternative medicinal agents in laboratory experiments, animal models, and clinical trials (Molinos et al., 2015; Pan et al., 2018). These therapeutic compounds demonstrate a range of phenomena that promote IVD regeneration and hinder degeneration. These include antioxidant, anti-inflammatory, anti-aging, anti-apoptotic, anti-catabolic, and anabolic actions.

Various small molecule drugs, such as naringin, cannabidiol (CBD), epigallocatechin gallate (EGCG), curcumin, icariin, resveratrol, berberine, and tofacitinib, were found to have an impact on the down-regulation of IL-1 and TNF-α in IVD cells, as observed in multiple *in vitro* investigations. Previous studies have reported that icariin, resveratrol, and EGCG possess inhibitory effects on NF-kB and p38/MAPK signaling pathways. As a result, these compounds are able to regulate inflammatory responses and impede the progression of degenerative cascades (Cao et al., 2016; Xu et al., 2018). Gefitinib, kaempferol, and berberine are further small compounds that selectively inhibit the NF-kB signaling pathway (Zhu et al., 2017; Pan et al., 2018; Lu et al., 2019). In contrast, rhein and uridine *In vitro*, the intracellular p38/MAPK signaling pathway was observed to be obstructed (Li et al., 2011; Liu et al., 2018).

The examination of anti-inflammatory medicines has demonstrated their efficacy in alleviating symptoms in individuals with IDD. Nevertheless, the precise mechanisms behind their potential anti-inflammatory and rejuvenating activities remain inadequately elucidated. In a study conducted by Li Z et al., the authors discovered the potential of etanercept and tofacitinib in preserving disc homeostasis within intervertebral disc bioreactors using preclinical disc organ culture models. This approach enabled the application of dynamic loading and facilitated nutrition exchange (Li et al., 2020). Etanercept was administered via intradiscal injection while simultaneously replenishing tofacitinib in the culture medium. The study employed immunohistochemistry as a method to evaluate the protein expression levels of IL-1β, IL-6, IL-8, and collagen II in IVD tissues. The expression of IL-1β, IL-6, IL-8, matrix metalloproteinase-1 (MMP1), and matrix metalloproteinase-3 (MMP3) in NP tissue, as well as IL-1β, MMP3, cyclooxygenase-2 (COX-2), and nerve growth factor (NGF) in annulus fibrosus (AF) tissue, was downregulated by the administration of etanercept and tofacitinib. Etanercept and tofacitinib have shown the capacity to counteract the proinflammatory and catabolic milieu in organ culture models of IDD.

IL-1β serves as the primary inflammatory component responsible for expediting the process of disc degeneration. Furthermore, there is an observed elevation in the levels of IL-1β within degenerated discs. In recent studies, it has been observed that luteolin, which belongs to the class of flavonoid glycosides, exhibits anti-inflammatory characteristics. Luteolin exhibited the ability to preserve cellular shape and suppress apoptosis in intervertebral disc NP cells treated with IL-1β. This was evidenced by a decrease in the production of cleaved caspase3 (Lin et al., 2019). Additionally, it demonstrated inhibitory effects on many inflammatory mediators, including nitric oxide (NO), prostaglandin E2 (PGE2), TNF-α, interleukin 6 (IL-6), COX-2, and inducible nitric oxide synthase (iNOS) in NP cells treated with IL-1β. The findings from mechanistic investigations demonstrated that luteolin exerted inhibitory effects on the NF-κB signaling pathway. Furthermore, it was shown that the regulation of luteolin in NF-κB signaling entailed the participation of Nrf2, as seen by the diminished inhibitory effect of luteolin on NF-κB signaling upon Nrf2 knockdown. This finding provides evidence that luteolin has the ability to stimulate the Nrf2/HO-1 signaling pathway, suggesting its potential as a therapeutic intervention for IDD.

Nonsteroidal anti-inflammatory medications (NSAIDs) are commonly employed as the primary therapeutic approach for alleviating pain symptoms by mitigating the inflammatory element of the pain pathway. COX-2 inhibitors belong to a category of NSAIDs that selectively inhibit the enzyme COX-2, resulting in the alleviation of inflammation and pain while minimizing gastrointestinal side effects compared to nonselective NSAIDs. NSAIDs have the potential to be utilized in conjunction with additional analgesics, including paracetamol and mild opioids (such as tramadol), in order to effectively address pain pathways at various stages. Tellegen AR et al. investigated the controlled release and biological effectiveness of celecoxib, a selective COX-2 inhibitor, from polyester amide microspheres in an *in vitro* setting (Tellegen et al., 2018). The experiment demonstrated that the release of celecoxib *in vitro* was prolonged for a period exceeding 28 days. This sustained release led to a notable reduction in inflammation, as indicated by the decreased production of PGE2. Additionally, the experiment revealed anti-catabolic effects in NP cells obtained from degenerative IVD, as evidenced by quantitative polymerase chain reaction (qPCR) analysis. Moreover, there is evidence suggesting that inflammation is involved, as indicated by the reduction in tissue levels of PGE2 and the decrease in immunopositivity of neural growth factor. These findings indirectly support the notion that the topical application of COX-2 inhibitors might effectively alleviate pain associated with intervertebral disc degeneration.

Rapamycin is a lipophilic antibiotic mainly used to prevent immune rejection after organ transplantation, owing to its immunosuppressive properties (Webster et al., 2006). It specifically inhibits the mammalian target of rapamycin (mTOR), a serine/threonine protein kinase that is crucial for cell growth and proliferation (Mossmann et al., 2018). Rapamycin is a specific inhibitor of mTOR and can also activate cellular autophagy, thereby protecting cells or tissues from further damage (Benjamin et al., 2011). Zuo Rui et al. constructed a mouse model with a degenerated cartilage endplate (CEP). Under the induction of rapamycin-induced autophagy, chondrocyte-like cells in CEP stem cells were protected from TNF-α-induced oxidative stress. The study also showed that rapamycin enhanced the Nrf2/Keap1 pathway, boosting the expression of antioxidant proteins (Zuo et al., 2019). Jinyu Bai et al. designed a reactive oxygen species (ROS)–clearing scaffold loaded with rapamycin (Rapa@Gel). After establishing a rat IVD injury model, Rapa@Gel treatment reduced the proportion of M1-like macrophages and alleviated inflammation in the damaged IVD tissue in rats, showcasing the IVD regenerative potential of rapamycin (Bai et al., 2020). Gao C. et al. (2018) reported that borrelidin treatment induced senescence in rabbit annulus fibrosus stem cells (AFSCs), but after rapamycin treatment, the gene expression of MMP-3, IL-1β, and TNF-α was downregulated in AFSCs. Furthermore, rapamycin could inhibit the multidirectional differentiation potential of AFSCs in a concentration-dependent manner, thus delaying IDD onset (Takahashi et al., 1999).

To date, numerous small-molecule drugs, including growth factors or cytokine inhibitors, have shown compelling and specific therapeutic effects in basic research (Wang et al., 2021; Wu et al., 2022; Chen et al., 2023b). However, none of these small-molecule drugs has yet been successfully applied in clinical settings to ameliorate IDD. There is also a lack of clinical studies on small molecule drugs for the treatment of IDD. Regarding this, the author has summarized the following reasons:

- The process of IDD is excessively complex and variable, involving factors such as inflammation, apoptosis, infection, mechanical stress changes, cell transplantation, and cell transformation. The intricate physiological and biochemical changes pose a challenge for a single drug to alter all physiological and biochemical activities during the degeneration process (Knezevic et al., 2021).
- The short half-life of various bioactive molecules within joints and IVD reduces the duration and effectiveness of drug concentration, preventing drugs from exerting a prolonged effect (Liu et al., 2023b).
- Despite the multitude of targets for the IDD process, critical targets are lacking. Most targets can only inhibit a specific degenerative process and cannot achieve comprehensive control, thus failing to influence the entire process of IDD (Guo et al., 2022).
- Cells from various sources within the IVD are overly complex, including NP cells, fibroblasts, and chondrocytes, all with different origins. The physiological activities of cells from different sources are significantly distinct, increasing the difficulty of drug treatment (Zhang et al., 2023).
- There are significant challenges in clinical trials. Clinical trials consume considerable time and financial resources, involving ethical issues and concerns about data validity and authenticity. Not every preclinically effective small molecule drug can be feasibly subjected to clinical trials”.

Hence, additional research is essential in identifying crucial targets, refining drug delivery systems, and conducting clinical trials. This necessitates collaborative efforts from experts within the industry.

### 3.2 Sirtuin family

**Figure 4:**
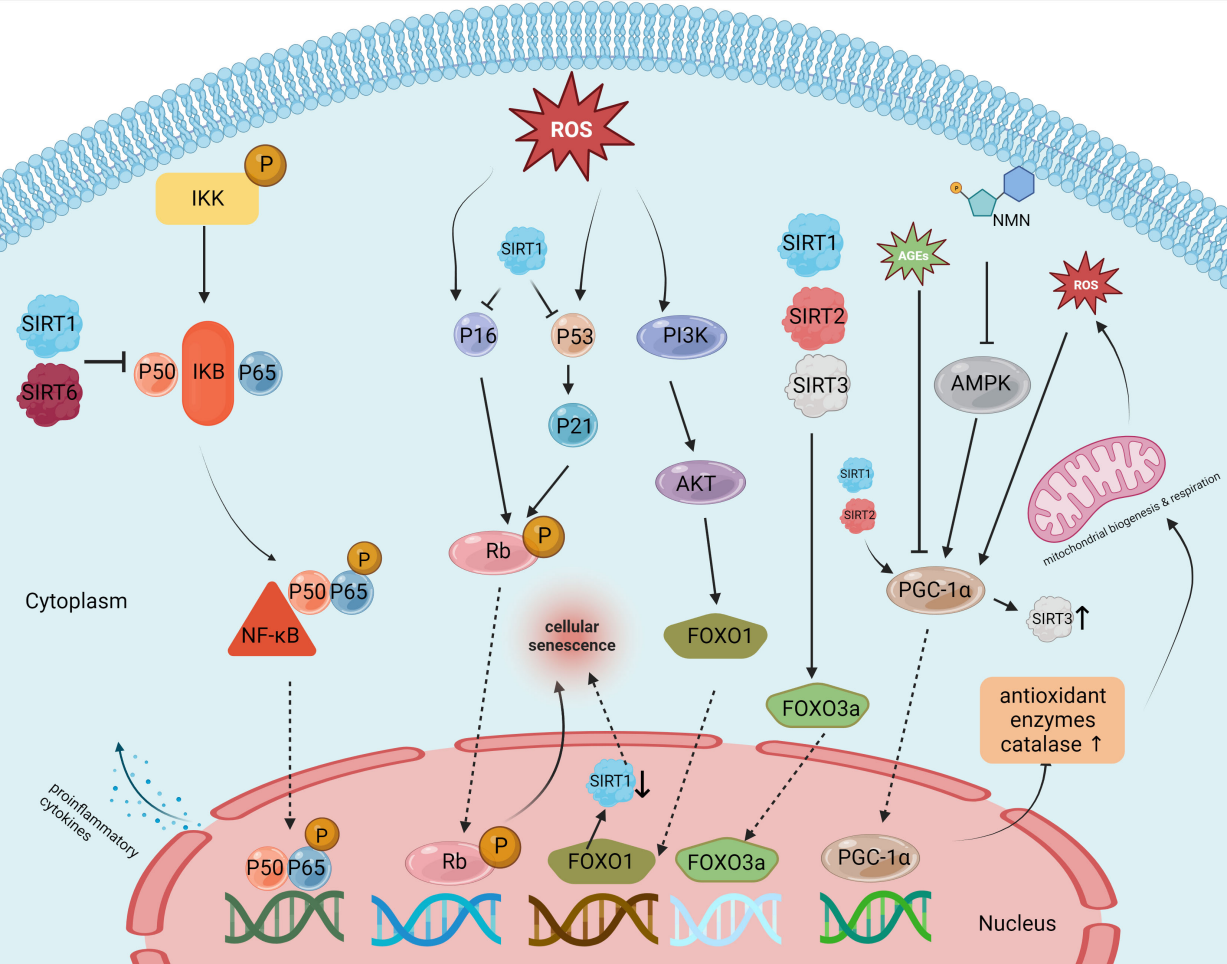
Targets and effects of sirtuin family members in various pathways of intervertebral disc degeneration.

Sirtuins are a family of protein deacetylases that are highly conserved and depend on nicotinamide adenine dinucleotide (NAD+) for their deacetylase activity. These proteins are pivotal in extending lifespan and delaying the onset of aging-related diseases (Wątroba et al., 2017). They are involved in various metabolic processes, including the regulation of inflammation (Mendes et al., 2017). Notably, several studies have identified the role of specific sirtuins—namely SIRT1, SIRT2, SIRT3, and SIRT6—in the occurrence and progression of IDD and the aging process of IVD cells (Cai et al., 2020; Ma et al., 2022; Hou et al., 2023). These sirtuins influence inflammation, oxidative stress, and mitochondrial dysfunction (Zhang et al., 2020) (**Figure 4**).

Among the sirtuin family members, SIRT1 is the most extensively studied protein and is associated with aging, cancer, and various degenerative diseases (Yang et al., 2022b). Existing studies indicate that SIRT1 has the potential to reduce inflammation, inhibit oxidative stress, and enhance mitochondrial function. These abilities make it a promising candidate for delaying the onset and progression of IDD (Ji et al., 2018). SIRT1 expression has been detected in NP cells. As IDD progresses, the expression level of SIRT1 mRNA has shown a notable decrease. Furthermore, *in vitro* tests have shown that activating SIRT1 with resveratrol, a known SIRT1 agonist, promotes NP cell proliferation while inhibiting apoptosis (Wuertz et al., 2011). Yi et al. (Yi et al., 2019) reported that NF-κB knockdown using P65-siRNA significantly reduces LPS-induced NP cell apoptosis and the expression of the pro-inflammatory factors TNF-α and IL-1β. The NF-κB signaling pathway is a crucial mediator in the inflammatory response of IDD. SIRT1 in IDD inhibits the NF-κB pathway and, consequently, reduces IL-1β-induced inflammation, thereby reducing NP cell apoptosis and ECM degradation. Further adding to SIRT1’s capabilities, Hao et al. (Hao et al., 2022) reported that the protein p300 could upregulate the expression levels of FOXO3 by binding to the promoter region of sirt1. This interrupts the Wnt/β-catenin pathway, contributing to reduced inflammation and delayed IDD progression. In addition to regulating the inflammatory response, oxidative stress resulting from excessive production of ROS can accelerate IDD through the modulation of signaling pathways such as NF-κB, MAPK, and PI3K/Akt pathways (Davalli et al., 2016). St-Pierre et al. reported that SIRT1 can inhibit this oxidative stress by inducing the deacetylation of PGC-1α, which leads to the overexpression of antioxidant enzymes, including manganese superoxide dismutase (Mn-SOD), which inhibits oxidative stress. Furthermore, SIRT1 deacetylates FOXO3a and translocates it to the nucleus, leading to upregulated expression of other antioxidant enzymes and peroxidases, protecting cells from damage caused by oxidative stress. During IDD progression, both the number and function of mitochondria in aging NP and annulus fibrosus cells tend to diminish, compromising their cellular function. Miyazaki et al. (Miyazaki et al., 2015) revealed that the administration of recombinant human SIRT1 (rhSIRT1) increased autophagy and reduced nutrient deprivation–induced mitochondrial apoptosis in cultured human NP cells. This suggests that rhSIRT1 might be an effective therapeutic approach for treating IVD-related diseases. In summary, SIRT1 presents a compelling case as a potential clinical target for delaying IDD and providing effective treatment for LBP.

Like SIRT1, SIRT2 is also ubiquitously found in the cytoplasm and nucleus across a variety of human tissues. Although it does not have as specific a regulatory role as SIRT1 in cellular functions, SIRT2 is closely linked to inflammation, oxidative stress response, and mitochondrial function. SIRT2 becomes upregulated under conditions of oxidative stress, leading to the deacetylation of FOXO3a and an increase in the expression of its target genes (p27 kip 1, MnSOD, and Bim). This chain of events subsequently reduces the production of reactive oxygen species (ROS) (Wang et al., 2019b). PGC-1α, a downstream molecule of SIRT2, acts as a transcriptional coactivator for numerous genes and plays a pivotal role in mitochondrial biogenesis, energy management, and cellular survival. It is also instrumental in clearing mitochondrial proteins of ROS (Halling and Pilegaard, 2020). Recent research has revealed that SIRT2 offers protection to annulus fibrosus cells from oxidative stress-induced apoptosis by regulating PGC-1α and inhibiting mitochondrial autophagy (Xu et al., 2019). A study by Yang et al. (Yang et al., 2019) showed that SIRT2 expression is markedly reduced in tissues affected by severe IDD. However, overexpressing SIRT2 in these degenerative NP cells notably inhibits the p53/p21 pathway, thereby slowing down tissue aging. Additionally, SIRT2 overexpression leads to an increase in the production of antioxidant enzymes SOD 1/2, mitigating oxidative stress in IVD cells. Given these findings, SIRT2 holds promise as a future target for preventing and delaying IDD.

In the realm of sirtuins research, SIRT3 stands out as the only member proven to extend human lifespan. Located primarily in the mitochondria, SIRT3 assumes a prominent role in mitochondrial function (Gao J. et al., 2018). Under stressful conditions, mitochondria may overexpress SIRT3, which then boosts the expression of the FOXO3a gene, essential for producing SOD2 and catalase. Advanced glycation end products (AGEs), which are associated with late stages of IDD, are known to induce oxidative stress and impair mitochondrial function (Liao et al., 2019). A study by Song et al. (Song et al., 2018) demonstrated that diminished SIRT3 functionality and reduced mitochondrial antioxidant capabilities are key factors in AGE-induced oxidative stress and the resulting apoptosis in human NP cells. Furthermore, nicotinamide mononucleotide has been shown to enhance SIRT3 functionality and thereby reduce apoptosis in NP cells through the AMPK-PGC-1α pathway (Martin et al., 2017). This suggests that SIRT3 plays a crucial role in preventing AGE-induced apoptosis in human NP cells and may be effective in delaying the progression of IDD by improving mitochondrial redox homeostasis.

Sirt6 has unique enzymatic activities, including both ADP-ribosyltransferase and NAD+-dependent deacetylase functions (Liu et al., 2021). Chen et al. (Chen et al., 2018) showed that levels of Sirt6 tend to decline in the NP cells of older individuals, while its overexpression can deter apoptosis in these cells. Another study by Jiang Hua et al. (Jiang et al., 2021) identified a significant upsurge in the levels of miR-338-3p in NP cells from patients with IDD. Injecting antagomir-338-3p attenuated the inhibitory effect of SIRT6, thus reducing cellular aging and apoptosis in NP cells. In summary, these findings suggest that SIRT6 could be a therapeutic target for delaying IDD progression by mitigating apoptosis in NP cells.

### 3.3 Cell therapy

During IVD, there is a steady decline in the population of healthy resident cells, which is accompanied by the progression of catabolic activity while tissue anabolism occurs (Vadalà et al., 2019). In order to promote the natural regenerative processes of degenerative IVD, stem cells can be obtained from diverse origins and afterward transplanted into afflicted host tissues. The concept of “stemness” is a subject of great interest, encompassing the transplantation of transdifferentiated somatic cells, induced pluripotent stem cells, and embryonic stem cells. Undifferentiated stem cells possess the capacity for self-renewal and proliferation, leading to the generation of specialized cells that replenish the population of cells within distinct tissues (Clouet et al., 2019). In this context, it is plausible that these entities possess the capability to release growth factors and cytokines, which serve the purpose of facilitating the functioning of existing cells and enticing or activating nearby progenitor cells. (Clouet et al., 2019; Vadalà et al., 2019). It has been proven that stem cells have been isolated from many tissues, including IVD (Kraus and Lufkin, 2017; Kraus et al., 2017).

Various types of candidate cells have been identified in the field of cell-based IVD therapy. These include NC cells, chondrocytes, MSCs, and NP cells. Some of these cell types have undergone preclinical and clinical investigations (Lufkin et al., 2022). Selection of cell types requires knowledge of disc development as well as understanding of maturation and degeneration-induced cellular changes (Pattappa et al., 2012; van den Akker et al., 2016; Binch et al., 2021). Promising approaches in the field of endogenous repair involve the activation of IVD repair cells, together with the administration of basic biological components such as microRNAs (miRNAs) (Henry et al., 2018; Clouet et al., 2019). Dong et al. (Dong et al., 2019)investigated the role of miR-640 in DDD and inflammation. Having confirmed that miR-640 was upregulated in both DDD tissues and cells, they found that this upregulation could be caused by an inflammatory environment. The application of TNF-α and IL-1β to cells led to a notable augmentation in miR-640 expression, which was facilitated through the NF-κB signaling pathway. In this inquiry, they aim to forecast the potential targets of miR-640 and substantiate the claim that one of its targets is the low-density lipoprotein receptor-related protein 1 (LRP1), which acts as an indirect inhibitor of NF-κB. Combining cell therapies with miRNAs has the potential to have a multiplier impact.

While cell therapy has demonstrated certain functional improvements in basic research when compared to control groups, its clinical translation has produced few satisfactory results. This is attributed to the intricate biological microenvironment of intervertebral discs, the complex mechanical stress on the spine, and the absence of a unified cell treatment strategy. Notable drawbacks include issues such as cell injection leakage, insufficient cell survival time, and ectopic ossification (DiStefano et al., 2022). Additionally, the regulatory measures and ethical concerns associated with cell therapy products derived from human sources pose significant challenges, consuming a substantial amount of time. Numerous studies have suggested that cell therapy achieves therapeutic effects by activating pathways through paracrine signaling molecules (Duan et al., 2023; Zhou et al., 2023). Consequently, extracellular vesicles (EVs) have emerged as a new focal point of research.

**Figure 5:**
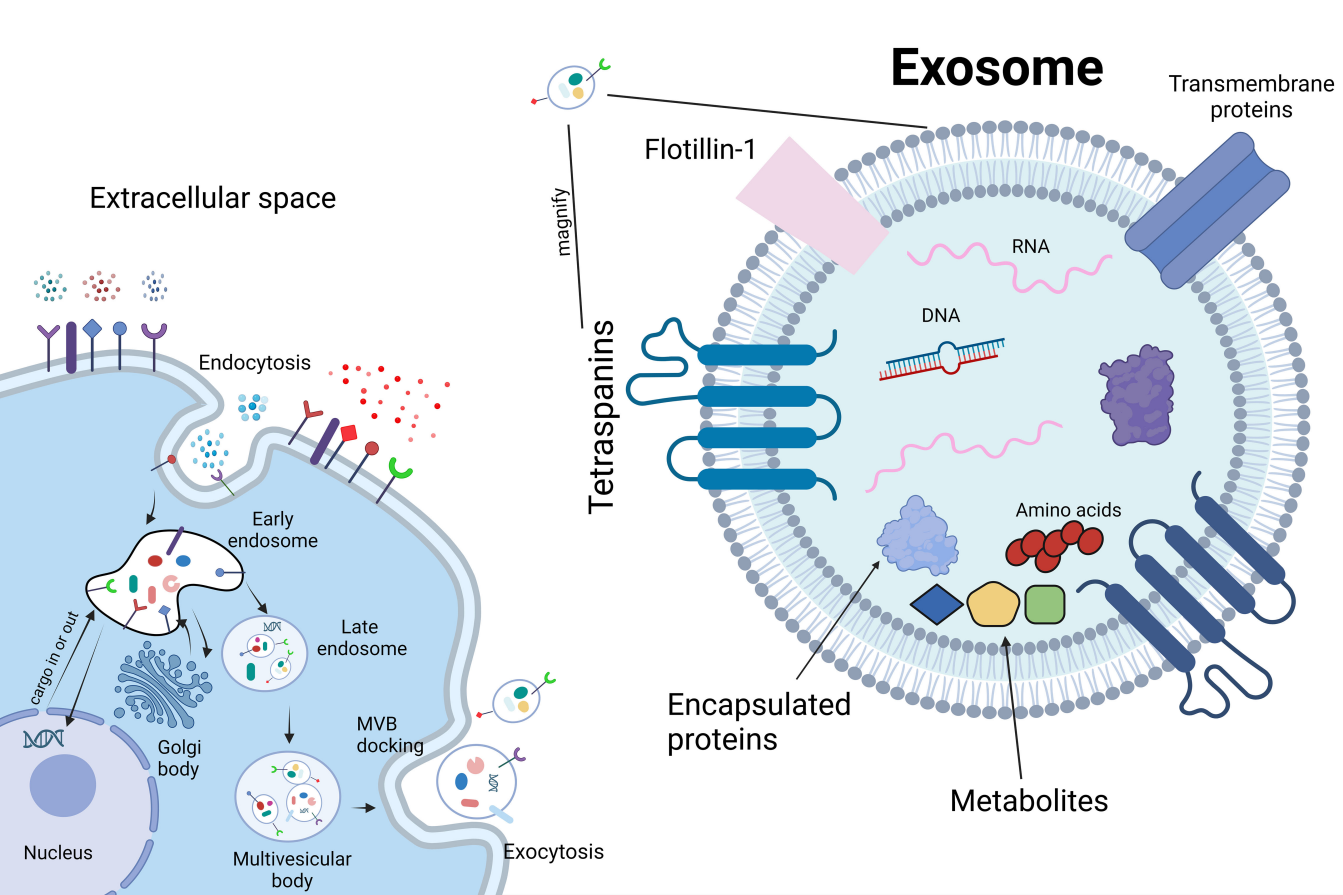
Mechanism of extracellular vesicle production.

EVs can be generated by nearly all cell types. These vesicles encapsulate various types of nanoscale particles, including lipids, nucleic acids, amino acids and their metabolites, diverse proteins, mRNA, short-chain non-coding RNA, and DNA (van Niel et al., 2022) (**Figure 5**). Exosomes, a subtype of EVs, play a significant role in intercellular communication. EVs act as a crucial means of conveying specific molecules efficiently from source cells to target cells (Buzas, 2023). The phospholipid bilayer on their surface exhibits recognition specificity, making EVs well-suited for precise biological marking and the transport of certain drugs (Jeppesen et al., 2023). EVs represent an emerging field of research in skeletal repair, with the majority of published studies emerging within the last five years (Yin et al., 2022; Ebata et al., 2023; Liu et al., 2023c). Documentation of the efficacy of EVs in various organs and systems exists, and in osteoarthritis (OA), it has been observed that EVs derived from mesenchymal stem cells (MSCs) can inhibit inflammation, downregulate the levels of IL-1β, IL-6, and IL-8, and increase extracellular matrix synthesis, effectively alleviating pain (Li et al., 2022). Although the mechanisms underlying OA and IDD are not entirely identical, evidence suggests that EVs could constitute a promising research direction for treating IDD (Liao et al., 2021).

In a study conducted by Hongxing Hu and colleagues (Hu et al., 2023), Evs derived from pre-treated MSCs under low oxygen conditions were injected into intervertebral disc tissues. The results demonstrated an enhancement in NP proliferation and increased production of proteoglycans and type I collagen. *In vivo* experiments further elucidated that Evs promote IVD regeneration through the transmission of miRNA-7-5p. In the research led by Zhiwei Liao and collaborators (Liao et al., 2019), it was observed that EVs derived from bone marrow mesenchymal stem cells (MSC-exos) can activate the AKT and ERK pathways, mitigating endoplasmic reticulum (ER) stress-induced cell apoptosis. Additionally, Hongyuan Xing (Xing et al., 2021) and his team integrated the extracellular matrix of thermosensitive cells into a hydrogel, combining it with EVs from adipose-derived mesenchymal stem cells (ADSC) (dECM@exo). This innovative approach, harnessing the biomechanical advantages of a hydrogel, consistently releases vesicles carrying nanoparticles that modulate matrix metalloproteinases (MMPs). This process aims to promote matrix synthesis, reduce degradation, and inhibit inflammation. Animal experiments validated its efficacy in maintaining the homeostasis of the early IVD microenvironment and improving the process of IDD.

While most *in vitro* experiments in existing studies have demonstrated the benefits of EVs for NP cells and IVD regeneration (Dai et al., 2023; Liu et al., 2023a), few studies have reported their efficacy on annulus fibrosus (AF) cells and cartilaginous endplate (CEP) cells (Yang et al., 2023). Future research could further enhance investigations into these two cell types. Additionally, there is a need to explore efficient methods for cultivating stem cells and Evs molecular carriers, among other considerations (Wu and Sun, 2021). In conclusion, Evs, as an emerging research focus, present unique advantages compared to traditional cell therapies. It is a subject worthy of deeper exploration and holds promise as a novel pathway in clinical treatment.

### 3.4 Biomaterials

#### 3.4.1 Hydrogel materials

The key to advancing the study of IVD regeneration and transformation, slowing down, and improving IDD processes, alleviating patients’ back pain, and enhancing their overall quality of life lies in the development of novel treatment strategies. NP cells have been identified as a promising therapeutic target. Numerous clinical studies have shown that autologous mesenchymal stem cells (MSCs) can differentiate into NP cells (Lv et al., 2022). A cutting-edge approach to treating IDD involves combining suitable biological materials with cells, which is a novel strategy. These materials not only counteract disc height reduction due to degeneration but also restore the biomechanical stability of the IVD (Yamada et al., 2022). Furthermore, they facilitate the controlled release of encapsulated cells or bioactive factors (Panebianco et al., 2020), thereby aiding in IVD repair and slowing down its aging process.

The engineering of these biomaterials is critically important and should meet certain criteria for maximum effectiveness. First, the biomaterials should replicate the gel-like and swelling properties of native NP cells (Chu et al., 2018). Additionally, these materials need to be permeable for nutrient exchange and hydrophilic to support the moist environment required for NP cell growth. Second, the biomaterials should encourage NP cell regeneration while inhibiting the ingrowth of vascular and neural tissues into the IVD. They should also allow for the effective release of either encapsulated cells or bioactive factors into the disc space. Third, these biomaterials must be stable, compact, and lightweight for straightforward clinical implantation (Fontana et al., 2015). Injectable hydrogels stand out as ideal candidates for such biomaterials (Cha et al., 2022). These hydrogels serve multiple functions: they can act as carriers for various cells and bioactive factors. The injectable hydrogel can easily mimic the biological properties of NP cells, and they can be introduced directly into the NP through minimally invasive injection techniques, minimizing tissue damage. Most importantly, the hydrogel can serve as a protective barrier for the biotherapy (cells or bioactive factors), preventing them from being directly exposed to the adverse cellular microenvironment in degenerated IVD (Tang et al., 2020). This enables the gradual release of therapeutic cells or factors to stimulate NP tissue regeneration without unwanted dispersion into surrounding areas. Directly injecting MSCs into the NP presents challenges due to conditions such as low glucose levels, high osmolarity, and low pH, which can adversely affect cell proliferation (Wuertz et al., 2008). Once injected, the hydrogel quickly ameliorates the biomechanical properties of the degenerated IVD and steadily releases biofactors that promote NP tissue regeneration (Xing et al., 2021).

Self-assembling peptide hydrogels offer a groundbreaking class of synthesized biomaterials that amalgamate the strengths of both natural and synthetic hydrogels for medical applications. These hydrogels possess several desirable traits, such as shear-thinning behavior, high biocompatibility, ECM mimicry, and tunable physicochemical properties, making them suitable and functional tools for addressing IDD. For instance, Bryant et al. engineered a dynamic multifunctional nanohybrid peptide hydrogel through layered self-assembly of peptide amphiphiles modified with enzyme-like biodegradable two-dimensional nanomaterials. This hydrogel not only offered excellent injectability but also possessed excellent anti-rejection and biodegradable properties (Conley et al., 2023). Huang Lin et al. combined the photocrosslinking of methacrylate chitosan (CSMA) through the Schiff base reaction between CSMA and aldehyde polyethylene glycol diacrylate (PEGDA) to form an injectable chitosan/PEG hydrogel (CSMA-PEGDA-L). Cell culture experiments showed that CSMA-PEGDA-L has low cytotoxicity. Imaging studies conducted using a rat animal model revealed that the hydrogel effectively delayed IDD progression through physical blocking (Huang et al., 2023).

Hyaluronic acid (HA) is a nonbranching high-molecular-weight polysaccharide naturally found in the ECM of various hydrated tissues such as articular cartilage, synovium, and IVD. It is essential for maintaining a high level of tissue hydration (Gupta et al., 2019). When dissolved in water, HA can generate high-viscosity solutions and can also be used clinically as an injectable viscoelastic supplement to replenish HA in the synovial fluid in osteoarthritis (Abatangelo et al., 2020). Sheida Jahanbekam et al. demonstrated using a rat model that combining HA and gelatin with deflazacort alleviates osteoarthritis (Jahanbekam et al., 2023). Additionally, Isa’s research group revealed that cross-linked HA hydrogels could suppress inflammation by downregulating specific receptors and neurotrophic factors in NP cells exposed to IL-1β in an *in vitro* inflammation model (Isa et al., 2015). This anti-inflammatory action seems to be related to HA’s ability to the binding of HA chains with CD44 receptors on cell surfaces, thereby preventing further inflammation in NP cells. Furthermore, the use of a 15% HA hydrogel loaded with MSCs in rat IVD resulted in cell proliferation, improved disc height, and alleviation of pain and inflammation (Mohd Isa et al., 2018).

IDD presents unique physical characteristics and pathological microenvironment, including inflammation and oxidative stress; however, effective self-repair is challenging in IDD because of its inflammatory and oxidative microenvironment. During IDD progression, an increased infiltration of M1 macrophages and secretion of pro-inflammatory cytokines. To address this, Cheng et al. (Cheng et al., 2022) designed a novel injectable composite hydrogel scaffold: an oligo (poly[ethylene glycol] fumarate)/sodium methacrylate (OPF/SMA) hydrogel scaffold loaded with dual drug/releasing poly (lactic-co-glycolic) acid (PLGA) microspheres containing IL-4 (IL-4-PLGA) and kartogenin (KGN-PLGA). The scaffold demonstrated excellent mechanical properties and low immunogenicity, along with a controlled drug release mechanism. Notably, IL-4-loaded PLGA microspheres (IL-4-PLGA) facilitate the transition of macrophages from the M1 to the M2 phenotype in the initial induction stage. Conversely, KGN-loaded PLGA microspheres (KGN-PLGA) yield enduring anti-inflammatory effects. Furthermore, the researchers delved into the potential mechanisms underlying immune modulation and the anti-inflammatory effects of the composite hydrogel scaffold. The scaffold promotes cell proliferation and viability *in vitro*. This composite hydrogel scaffold maintains mechanical strength, regulates the local inflammatory microenvironment, and facilitates continuous repair of the NP tissue through sequential release of drugs *in vivo*. Chen et al. constructed a hydrogel combining HA and polyamide-based hydrogel (PAMAM) with siSTING-RNA to target the STING-NF-kB signaling pathway, a significant contributor and critical factor in IDD. This approach is superior to traditional siRNA therapy and overcomes limitations such as low cellular uptake, short half-life, and rapid siRNA degradation. Tests in a puncture-induced rat model of IDD confirmed that this siSTING-loaded hydrogel significantly alleviated IVD inflammation and decelerated IDD by prolonging STING knockdown (Chen et al., 2022a). In subsequent studies, they further incorporated P65 siRNA into a hydrogel modified with phenylboronic acid-acid-functionalized G5 PAMAM dendrimers (siRNA@G5-PBA@Gel), offering sustained drug release for over 28 days *in vitro* and *in vivo*. Combining this approach with cellular therapy can significantly enhance IVD regeneration abilities (Chen et al., 2023a). In summary, injectable hydrogels are promising for IDD treatment, mimicking the biological properties of NP cells and enabling the incorporation of various cells or biofactors. This multi-faceted approach enhances therapeutic efficacy, offering a credible pathway to delay IDD progression.

#### 3.4.2 Other biomaterials

**Figure 6:**
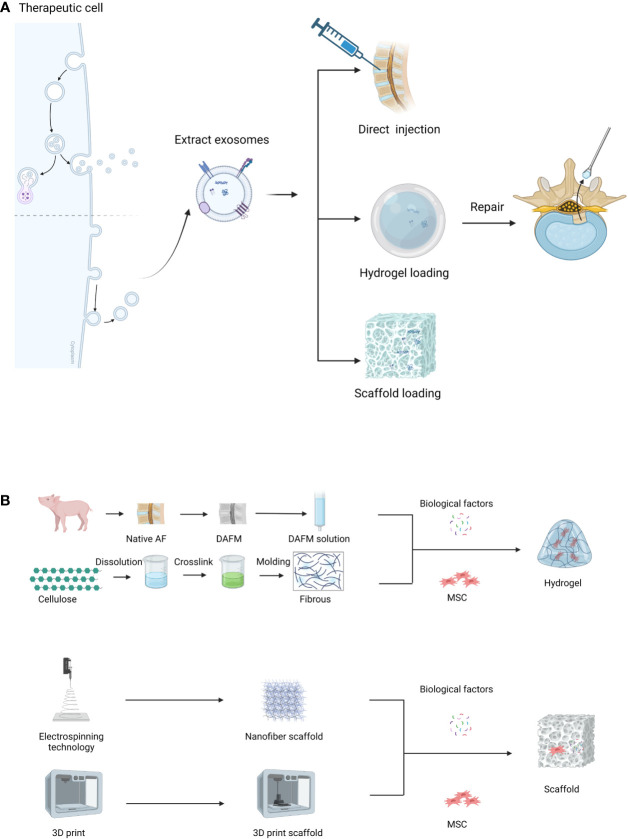
**(A)** Three routes to EVs therapy, **(B)** Preparation process of hydrogel or scaffold.

The consensus is that IVD exhibits minimal intrinsic self-repair capabilities following injury. While various supportive materials are available, each comes with its limitations (**Table 2**). Current bioengineering strategies endorse the integration of cells or factors promoting disc regeneration into implant materials, significantly enhancing the repair functionality of these implants (**Figure 6**). This paper reviews several representative biomaterials used in the recent treatment of IDD, summarizing their characteristics (**Table 3**).

**Table 2:** Various IVD support materials.

Types	advantage	disadvantage
Artificial Disc	good structure and function, stabilization	high-risk, immunoreaction
Disc Replacement Materials	low-risk, alleviating pain	unsteadiness, dislodgement of implantation materials
Metal Spacers	good stability, long durability	ankylosis, metal sensitivity
Polymeric Spacers	handiness, no anaphylaxis, elasticity	poor durability, need more research
Biological Spacers	high compatibility, helps regenerate and repair intervertebral discs	expensive, need more research
3D-Printed Spacers	high adaptability, individuation	expensive, need more research

**Table 3:** Recent representative biomaterials in the treatment of IDD.

Types of biological materials	Mode of administration	Subject	Effect	Refs.
multifunctional gelatin methacrylate (GelMA) microspheres	ex vivo and *in vivo*	Rats NP cells	improved the release kinetics of TGFβ3, effectively inhibited inflammatory, promoted the secretion of ECM	(Zhu et al., 2023)
NO-releasing micellar nanoparticles	ex vivo	Rats	efficiently eradicate C. acnes pathogens, inhibit the inflammatory response and osteoclast differentiation	(Tao et al., 2022)
injectable composite hydrogel scaffold	ex vivo and *in vivo*	Rats NP cells	increase in the proportion of M2 macrophages, higher expression levels of type II collagen, long-term anti-inflammatory effects	(Cheng et al., 2022)
injectable bioorthogonal hydrogel (BIOGEL)	ex vivo and *in vivo*	Rats NP cells	potentiated histological repair, functional recovery	(Luo et al., 2023)
aligned core-shell nanofibrous scaffolds loaded with TGFβ3 and IBU	ex vivo and *in vivo*	Rats NP cells	good anti-inflammatory ability enhance ECM formation and maintain the mechanical properties of IVD	(Han et al., 2022)
injectable collagen scaffold with ASC	ex vivo	Sheep model	less degeneration-specific features, stabilization of the disc height	(Friedmann et al., 2021)
injectable composite hydrogel, Mel-MBG/SA	ex vivo and *in vivo*	Rats NP cells	alleviate IL-1β-induced oxidative stress, relieve inflammation associated with IDD pathology	(Wu et al., 2023)
OxAlg with MBs combine FibGen hydrogels	ex vivo	Bovine AF cells	minimized AF cell apoptosis and retained phenotypic gene expression, biomechanically stable, promote ECM synthesis	(Panebianco et al., 2022)

TGFβ3, transforming growth factor β3; ECM, extracellular matrix; NO, nitric oxide; C.acnes, Cutibacterium acnes; IBU, ibuprofen; ASC, adipose-derived stem cells; Mel, melatonin; OxAlg, oxidized alginate; MBs, microbeads; FibGen, genipin-crosslinked fibrin.

### 3.5 Clinical trials

At present, recent research on the treatment of IDD primarily centers on diverse modalities involving cells, growth factors, small molecule drugs, and combinations with or without biomaterials. Numerous *in vitro* and preclinical studies have demonstrated the ability of these approaches to induce the regeneration of NP cells through various mechanisms, thereby facilitating the self-repair of IVD. Preliminary results from some clinical trials are also available. The clinical trials focusing on IDD degeneration treatment conducted in the past five years total 12, as summarized in **Table 4**.

**Table 4:** Clinical trials on the treatment of IDD within 5 years.

Reference	Clinical Trial Number	Type of study	Year	Design and interventions	Number of patients	Observation duration	Analysis Variables	Deliverables
(Clavo et al., 2021)	NCT00566007	randomized double-blind controlled trial	2021	ozone	19	78 months	Safety, days and costs of hospitalization	fewer inpatient days and lower costs。
(Beall et al., 2021)	NCT03709901	prospective, multicenter, blind, randomized clinical trial	2021	Disc Tissue Allograft	218	12 months	VASPI ODI AEs	Clinically meaningful improvement in both VASPI and ODI, no persistently symptomatic AEs
(Bråten et al., 2019)	NCT02323412	double blind, parallel group, placebo controlled, multicentre trial.	2019	750 mg amoxicillin	180	12 months	RMDQ	three months of treatment with amoxicillin did not provide a clinically important benefit compared with placebo
(Amirdelfan et al., 2021)	NCT01290367	multicenter, randomized, controlled study	2020	STRO-3+MPCs+HA	100	36 months	Radiographically evaluated VASPI ODI AEs	Clinically meaningful improvement in both VASPI and ODI, no persistently symptomatic AEs
(Mazza et al., 2020)		multicentric open label study	2020	a novel Hydrogel (HYADD4-G)	23	6 months	VAS, MRI, RMDQ, EQ-5D	Decrease in VAS, RMDQ scores, EQ-5D scores rise, Improvement in Imaging Indicators
(Noriega et al., 2021)	NCT01860417	randomized controlled trial	2020	Allogeneic Mesenchymal Stem Cells	24	3 years	VAS, MRI, Oswestry Disability Index	Pfirrmann graded quantitative improvement, early pain improvements and the Oswestry Disability Index improvements
(Cheng et al., 2019)		randomized controlled trial	2019	autologous platelet-rich plasma	29	5-9 years	pain, function, satisfaction, and need for surgery	demonstrated statistically and clinically significant improvements in pain and function
(Ercalik and Kilic, 2020)		prospective, double-blinded, randomized controlled trial	2020	Ozone, Steroid	65	6 months	VAS, ODI	intradiscal ozone injection alone was sufficient to treat low back and leg pain and that periforaminal steroid injection does not provide additional benefit
(Lee et al., 2023)	NCT05011474	prospective study randomized clinical trial	2023	Matrilin-3-Primed Adipose-Derived Mesenchymal Stromal Cell Spheroids	8	6 months	VAS, ODI, MRI	Improvement in VAS, ODI scores, no change in Pfirrmann classification
(Akeda et al., 2023)		retrospective analysis randomized clinical trial	2023	Intradiscal Platelet-Rich Plasma-Releasate Injection	15	12 months	MRI, imaging manifestations	Radiographic parameters showed no significant changes, significantly improved LBP and LBP-related disability
(Zhang et al., 2022)	CTR1900024268	prospective clinical study	2022	Autologous Platelet-Rich Plasma	33	12 months	IVD, MRI	relieve pain sensation and improve lumbar function
(Atluri et al., 2022)	NCT04559295	Prospective, open-label, nonrandomized, parallel-controlled, 2-arm exploratory study	2022	Autologous Bone Marrow Mesenchymal Stem Cells	80	12 months	ODI, EQ-5D, MRI, GMH, GPH, MCID, NRS-11	Significant improvement was achieved in functional status measured by ODI, pain relief measured by NRS-11, and other parameters measured by EQ-5D-3L, GMH, and GPH,

VASPI, Visual Analog Scale of Pain Intensity; ODI, Oswestry Disability Index; AEs, Adverse events; RMDQ, Roland-Morris Disability Questionnaire; MPCs, mesenchymal precursor cells; HA, hyaluronic acid; EQ-5D, EuroQol-5 Dimension; GMH, Global Mental Health; GPH, Global Physical Health; MCID, minimal clinically important differences; NRS, Numeric Rating Scale.

## 4 Conclusions and perspectives

This review introduces the relationship between the occurrence of LBP and IVD infections, emphasizing the role of inflammatory responses in the progression of IDD and the development of LBP. The main treatment modalities for clinical management of LBP currently focus on pain-relieving medications and surgical interventions with the aim of providing immediate relief from severe pain. However, these approaches do not address the progression of IDD, resulting in recurrent LBP and significant impairment in daily life. The burden on healthcare resources and society is substantial. Contemporary medical practice now emphasizes personalized treatment, with a focus on alleviating the progression of IDD and promoting IVD regeneration and recovery rather than solely addressing pain relief. Consequently, there has been extensive research on various novel treatment targets and approaches as basic medicine integrates with clinical medicine.

Inflammation is an important factor in IDD and LBP. Most IDD is initially caused by sustained pressure on the fibrous rings and endplates, leading to age-related IDD. Therefore, it is believed that most IDD is the result of sterile inflammation. However, the issue of whether bacterial infection accompanies or is secondary to IDD remains highly controversial. While some reports suggest that symptoms in IDD patients improve after antibiotic treatment, other reports indicate that the effectiveness of antibiotic treatment does not meet the criteria for clinical effectiveness in IDD patients. The variation in effectiveness may be attributed to factors such as the type of antibiotic used, the dosage, and the duration of administration ^[113]^. It is important to note that this paper does not address the mechanism and pharmacological effects of antibiotics in the treatment of IDD, nor does it propose a definitive treatment for IDD in cases of clinical septic inflammation. These areas require further research and exploration by scholars.

In recent years, the focus of modern clinical treatment has shifted towards personalized care, specifically in regulating disease progression from a microscopic perspective. As a result, suppressing Inflammation, inducing NP cell regeneration, improving the cellular microenvironment, and slowing down IDD have become key areas of research interest. Significant progress has been made in the study of various cell therapies, small molecular drugs, and bioengineering materials (Sakai and Grad, 2015), with cell and biofactor therapies showing promise in inhibiting inflammatory signaling pathways and promoting NP cell regeneration. To effectively control potential targets, such as sirtuins and miRNAs, which are not yet clinically applicable but have potential for slowing down the process of IDD, it is crucial to use relevant biological factors or drugs. However, utilizing these targets requires more in-depth and extensive trials conducted by experts in the field. Furthermore, an ongoing challenge is achieving effective long-term release of therapeutic cells or factors in the IVD(Lazarus et al., 2021). With the advancement of bioengineering scholars have investigated a biomaterial akin to medullary tissue - hydrogel. Hydrogels have emerged as potential solutions, as they can carry diverse drugs, cells, and biofactors. Nevertheless, hydrogels are currently made in a variety of ways, and a large range of cells or drugs can be coupled with hydrogels. Most notably, all hydrogel studies have only shown efficacy in rat models, but have never been examined in a sheep model, which is more structurally similar to the human spine (Wang et al., 2019a). In contrast, several other biosupport materials have been validated in sheep models. As a result, more standardised methods for the creation of effective hydrogel materials must be validated in order to determine their clinical efficacy. The purpose of this paper is to provide fresh insights into the clinical management of inflammation-induced IDD and LBP by detailing numerous drugs, materials, methods and clinical trials that have emerged in recent years that slow down the IDD process and promote IVD regeneration. Then again, it is important to note that many of the studies mentioned are still in the preliminary stages and require further exploration and research by experts and scholars in the field.

## Author contributions

SY: Writing – original draft, Writing – review & editing. SJ: Writing – original draft, Writing – review & editing. SW: Funding acquisition, Project administration, Supervision, Validation, Writing – review & editing. FJ: Funding acquisition, Project administration, Supervision, Validation, Writing – review & editing.

## Glossary

**Table:**

LBP	low back pain
IVD	intervertebral disc
IDD	intervertebral disc degeneration
NP	nucleus pulposus
TNF-α	tumor necrosis factor-alpha
IL-1β	interleukin-1 beta
IL-1α	interleukin-1 alpha
NF-κB	nuclear factor-kappa B
DDD	degenerative disc disease
ECM	extracellular matrix
HNP	herniated nucleus pulposus
CONS	coagulase-negative staphylococci
NLRP3	nod-like receptor protein 3
MRSA	methicillin-resistant Staphylococcus aureus
mTOR	mammalian target of rapamycin
CEP	cartilage endplate
CEPCs	cartilage endplate stem cells
AFSCs	annulus fibrosus stem cells
RMDQ	Roland-Morris Disability Questionnaire
CBD	cannabidiol
EGCG	epigallocatechin gallate
MMP1	matrix metalloproteinase-1
MMP3	matrix metalloproteinase-3
COX-2	cyclooxygenase-2
NGF	nerve growth factor
AF	annulus fibrosus
NO	nitric oxide
PGE2	prostaglandin E2
TL-6	interleukin 6
iNOS	inducible nitric oxide synthase
NSAIDs	nonsteroidal anti-inflammatory medications
qPCR	quantitative polymerase chain reaction
ODI	Oswestry Disability Index
NAD+	nicotinamide adenine dinucleotide
FOXO3 OR FOXO3a	Forkhead box O3
ROS	reactive oxygen species
Mn-SOD	manganese superoxide dismutase
rhSIRT1	recombinant human SIRT1
AGEs	advanced glycation end products
NMN	nicotinamide mononucleotide
ADP	adenosine diphosphate
PLGA	poly (lactic-co-glycolic).
